# Thermophysiology and Cognitive Performance of Live-Line Workers in High-Temperature and High-Humidity Environments

**DOI:** 10.3390/ijerph22030387

**Published:** 2025-03-07

**Authors:** Shengwei Wang, Xiaohong Gui, Li Ding

**Affiliations:** 1 School of Emergency Management & Safety Engineering, China University of Mining and Technology (Beijing), Beijing 100083, China; 2 School of Biological Science and Medical Engineering, Beihang University, Beijing 100191, China

**Keywords:** high-temperature and humidity, thermal sensation, occupational safety, skin temperature, heat stress

## Abstract

Live-line workers’ physiological and psychological conditions are significantly affected when operating in high-temperature and high-humidity environments, influencing both work efficiency and safety. Fifteen participants, wearing high-voltage-shielding clothing, were tested in a simulated environmental chamber at temperatures of 23 °C, 32 °C, and 38 °C, and relative humidities of RH 30%, RH 50%, and RH 75%. The experiment involved walking at a speed of 5 km/h for 75 min., during which the participants’ skin temperature, core temperature, thermal sensation, heart rate, blood oxygen level, sweat rate, and cognitive performance were measured. The results indicated a marked increase in both core and skin temperatures with rising temperature and humidity levels. At 38 °C/RH 75%, the core temperature reached 38.39 °C, and the average skin temperature was 36.8 °C. Significant differences in skin temperature were observed across different body regions (*p* < 0.05), with this disparity decreasing as the temperature increased. Heart rate, blood oxygen level, and sweat rate also exhibited significant differences across varying conditions (*p* < 0.05). Specifically, heart rate and blood oxygen level increased with higher temperature and humidity, while blood oxygen levels decreased as the environmental temperature and humidity increased. In addition, as temperature and humidity levels rose, the participants’ error rate and average response time in cognitive tasks increased. The negative impact of temperature and humidity on performance efficiency and accuracy was more pronounced in complex cognitive tasks. The study further found that thermal sensation voting (TSV) remained within the range of −0.5 to +0.5, with the average skin temperature in the thermal comfort zone ranging between 33.4 °C and 34.1 °C. It is recommended that the environmental temperature in high-humidity conditions be maintained between 20.8 °C and 25.8 °C. Our findings provide a theoretical foundation for the development of personal protective equipment for live-line workers.

## 1. Introduction

Over the past few decades, global climate change has led to an increasing frequency of extreme heat and heatwave events, severely threatening the safety of outdoor workers’ lives [1,2,3]. The frequency and intensity of extreme heat events have significantly increased globally, coupled with higher humidity during the summer, resulting in many excess deaths [4,5,6,7,8,9]. Typically, living environments above 35 °C and working environments above 32 °C are defined as high-temperature environments, while environments with relative humidity (RH) above 60% are defined as high-humidity environments [7,8,9]. Hot and humid environments impose a burden on human comfort, psychological health, and labor productivity [10,11]. This poses a threat to the safety of outdoor workers in industries such as electricity, firefighting, and construction, particularly those involved in live-line work during summer electrical maintenance. Live-line work refers to methods of conducting maintenance and testing on high-voltage (above 10 kV) electrical equipment without shutting down power, and it can be categorized into live-line testing, live-line inspection, and live-line maintenance, among other activities [12]. According to workers from the State Grid Corporation of China, due to the extended preparation time and operational processes, they typically perform 1–1.5 h of work during the hottest part of the day, around noon. Live-line workers must wear high-voltage-shielding clothing, which differs from the ordinary workwear worn by electricians: high-voltage-shielding clothing (with shielding efficiency typically ranging from 40 to 60 dB) effectively protects against the harmful effects of electric fields, but their breathability is poor, and they lack effective cooling methods, leading to severe heat stress issues for workers. Therefore, investigating the changes in physiological parameters and cognitive abilities of live-line workers under high-temperature and high-humidity conditions is crucial for developing protective measures to ensure their safety and work efficiency.

Many scholars have conducted extensive research on human thermal responses and cognitive testing in hot environments. Li et al. [13] tracked changes in field labor productivity and outdoor heat stress at different times during a 54-day measurement period in the summer of 2014. Ye et al. [14] found that skin temperature, ECG, and EEG are sensitive to environmental temperature and thermal perception, while Hayden et al. [15] emphasized the psychological and physiological burdens of prolonged exposure to hot and humid environments. Wu et al. [16] highlighted the influence of temperature on skin temperature and cognitive functions, although their studies had limitations such as low temperature ranges and neglect of humidity’s impact. Cui et al. [17] found that high temperatures could reduce cognitive performance. Some researchers have drawn the same conclusion, stating that high temperatures reduce attention and alertness [18,19,20,21,22]. However, other researchers have reached opposite conclusions [23,24,25]. Most of these studies did not consider the impact of humidity variation on cognition, focusing solely on the effects of temperature. Furthermore, most of the above studies focus on populations such as construction workers, pilots, office workers, and soldiers, with little research on the thermophysiology and cognition of live-line workers wearing shielding clothes.

Based on this, the purpose of this study is to investigate the thermal stress caused by high-voltage-shielding clothing in high-temperature and high-humidity environments during live-line work. Our research specifically analyzes how temperature and humidity affect the thermal physiology and cognitive functions of workers performing live-line operations. By systematically examining the human thermal responses of workers wearing high-voltage-shielding clothing under various environmental conditions, the study aims to explore the effects of different temperature and humidity on physiological indicators such as core temperature, skin temperature, heart rate, and sweat volume. Ultimately, this research seeks to identify the physiological characteristics of live-line workers in these extreme conditions, to provide a basis for proposing effective cooling measures to improve occupational safety and work efficiency, and to provide a theoretical basis for the development of personal protective equipment.

## 2. Methods and Materials

### 2.1. Experimental Equipment

**Figure 1:**
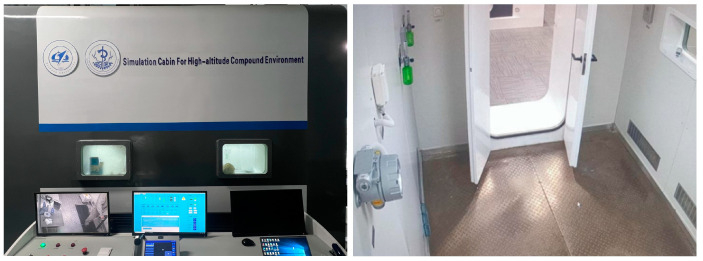
The high-altitude composite environmental simulation chamber.

The experiment was conducted in the high-altitude composite environmental simulation chamber at Beihang University (3 m × 2 m × 3 m, Guizhou Fenglei Aviation Ordnance Co., Guizhou, China), as shown in Figure 1. The temperature control of the chamber uses the HFW series precision temperature and humidity control system, with a temperature range of −10 °C to 60 °C and a humidity range of 20% to 95%; the temperature control precision is ±0.2 °C, and the humidity control precision is ±5%. The skin and oral temperatures of the participants were measured using iButton temperature sensors from Dallas Semiconductor (Analog Devices Inc. (ADI), Beijing, China), with the sampling frequency set to 1 per minute. The sensor’s temperature range is −20 to 85 °C, with an accuracy of ±0.1 °C. The heart rate and blood oxygen levels of the participants were measured using a PC-3000 monitor (Likang Biomedical Technology Holdings Limited, Shanghai, China). The heart rate range is 15–350 bpm, with an accuracy of ±2 bpm. The blood oxygen measurement range is 0–100%, with an accuracy of ±2%, with measurements taken every 5 s. The sweat of the participants was measured using a Mettler Toledo precision scale (METTLER TOLEDO International Ltd., Shanghai, China), with a measurement range of 0–100 kg and an accuracy of ±1 g. A treadmill was used to simulate the labor intensity of live-line workers.

### 2.2. Participants

This study recruited 15 healthy male university students as participants (mean age: 25.2 ± 1.7 years, mean height: 175.2 ± 2.6 cm, mean weight: 68.4 ± 5.1 kg, mean body mass index (BMI): 22.3 ± 1.9 kg/m^2^). None of the participants had a history of heart disease, physical disabilities, or other restrictions, and all met the experimental requirements. Before the experiment, all participants refrained from consuming alcohol, or caffeine, or engaging in intense physical exercise for at least 24 h. Additionally, under all experimental conditions, participants were required to wear standardized clothing, and complete an equal intensity of work to simulate the working conditions of live-line operators, which consisted of undergarments and live-line work shielding clothes worn by live-line workers. The thermal resistance of the clothing was approximately 1.12 clo. All participants were familiar with the experimental protocol and provided written informed consent to participate in the study, which was approved by the Committee on Biological and Medical Ethics of Beihang University (BM20240199).

### 2.3. Experimental Program

The experiments were conducted randomly under six environmental conditions, during the period when the participants’ mental states were at their best, with at least a one-day break between experiments. The temperature and humidity inside the chamber were set according to the Chinese high-temperature weather warning standard as follows:

(1) Investigating the effects of different temperatures (23 ± 0.2 °C, 32 ± 0.2 °C, and 38 ± 0.2 °C) in a high-humidity (RH 75 ± 5%) environment on the physiological parameters of individuals wearing shielding clothing.
(2) (Investigating the effects of different humidity levels (RH 30 ± 5%, RH 50 ± 5%, and RH 75 ± 5%) in a high-temperature (38 ± 0.2 °C) environment on the physiological parameters of individuals wearing shielding clothing.
(3) Investigating the changes in physiological parameters of individuals wearing shielding clothing and regular work attire (e.g., electricians’ typical short-sleeve shirts and long pants) under high-temperature and high-humidity conditions (38 ± 0.2 °C, RH 75 ± 5%).

### 2.4. Experimental Procedure

(1) Pre-experiment Preparation

At least 30 min before the experiment, the participants arrived at the experimental site 30 to undergo heat adaptation. The room temperature during the adaptation period was set to the starting temperature of the experimental conditions. During this period, the participants changed into experimental clothing and wore temperature sensors. Basic information such as name, age, height, and body weight was recorded.

(2) Post-adaptation Measurements

After the adaptation period, the participants were connected to a monitoring device (PC-3000) to measure heart rate and blood oxygen levels. Basic environmental parameters, including air temperature, humidity, and wind speed, were measured and recorded. Once the climate chamber’s temperature and humidity stabilized, the participants’ weight was measured for the first time, and a participative questionnaire was filled out. After a 10 min rest, a second weight measurement and questionnaire were completed.

(3) Simulated Work Intensity Experiment

After the 10 min rest, the participants exercised on a treadmill at a speed of 5 km/h to simulate the work intensity of electrical workers. They rested for 5 min after every 10 min running session, completing a total of four sessions. At the end of each running session, a questionnaire survey and weight measurement were conducted. (The experimental duration was determined through a pre-experiment. In the pre-experiment, over 90% of the participants had to terminate the experiment around the 70 min mark.)

(4) Post-exercise Rest and Data Collection

**Figure 2:**
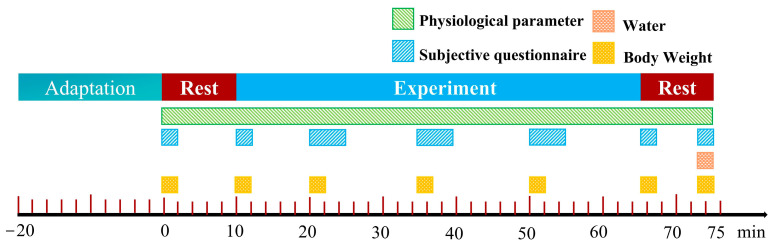
Experimental procedure.

After completing the running, the participants rested for 10 min and completed a final questionnaire and weight measurement. A total of seven questionnaires and weight measurements were collected. The experimental procedure is shown in Figure 2. The experiment could be stopped at any time by the operator if the participant felt unwell or if their heart rate exceeded 160 bpm. Each person participated in only one experiment for each working condition and did not repeat the experiment.

### 2.5. Parameter Measurement

**Figure 3:**
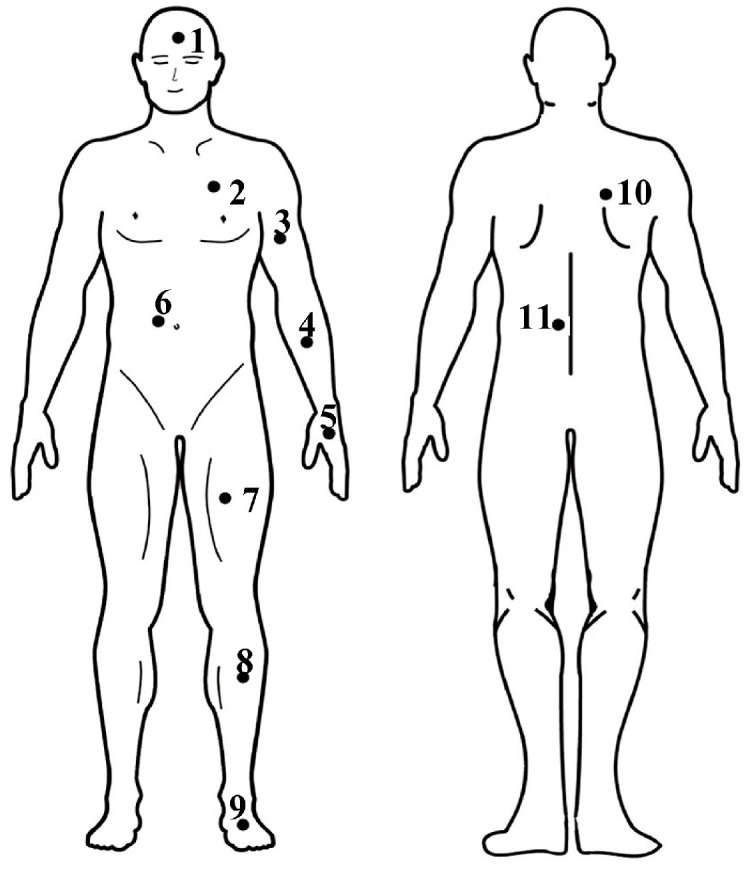
Location of simulation chamber experiments and temperature measurement points.

This study measured various physiological parameters of the participants, including oral temperature, local skin temperature, blood oxygen levels, and heart rate. Skin temperature was measured using the 11-point method, where numbers 1 to 11 represent the forehead, chest, upper arm, forearm, back of the hand, abdomen, thigh, calf, dorsum of the foot, back, and waist (Figure 3). The weighted average skin temperature was calculated using Equation (1) [26,27]. Sweating was measured using the weight differential method. The core temperature was calculated by adding 0.3 °C to the oral temperature [28,29].

(1) $$\begin{array}{l} T_{sk}=0.06T_{\mathrm{head}}+0.09T_{\mathrm{chest}}+0.09T_{\mathrm{abdomen}}+0.09T_{\mathrm{back}}+0.09T_{\mathrm{waist}}+0.07T_{\mathrm{upper}\;\mathrm{arm}}\\ +0.07T_{\mathrm{forearm}}\;+0.05T_{\mathrm{hand}}+0.19T_{\mathrm{thigh}}+0.13T_{\mathrm{calf}}+0.07T_{\mathrm{foot}} \end{array}$$

where *T_sk_* is the average skin temperature, °C; *T*_head_, *T*_chest_, *T*_abdomen_, *T*_back_, *T*_waist_, *T*_upper arm_, *T*_forearm_, *T*_hand_, *T*_thigh_, *T*_calf_, *T*_foot_ are the skin temperatures of the head, chest, abdomen, back, waist, upper arms, forearms, hands, thighs, calves, and feet, respectively.

The comprehensive heat stress index (CHSI, Equation (2)) was calculated based on heat storage, the increase in body temperature, the sweat rate, and the increase in the heart rate [30].

(2) $$\mathrm{CHSI}=0.01\Delta P+\Delta T_{b}+0.1\Delta W+S$$

(3) $$\Delta W=W_{2}-W_{1}$$

(4) $$S=3.486T_{b}$$

where Δ*P* is the increase in heart rate, in times/min; *T*_b_ is the body temperature, in °C; Δ*W* is the human sweating rate, in g/min; *W*_1_ is the body weight before the experiment, and *W*_2_ is the body weight after the experiment, in kg; *S* is the body’s heat storage capacity, in kJ/kg; and 3.486 is the specific heat capacity of human tissue, in kJ/(kg·°C).

(5) $$T_{b}=0.8T_{c}+0.2T_{\mathrm{sk}}$$

where *T*_c_ is the core temperature, °C.

The thermal sensation of the participants was assessed using a nine-point scale, as defined by ISO 10551 [31], ranging from extremely hot (+4), hot (+3), warm (+2), slightly warm (+1), neutral (0), slightly cool (−1), cool (−2), and cold (−3) to extremely cold (−4). Before the participants completed the questionnaire for the first time, the experimenter provided clear explanations for each item.

**Figure 4:**
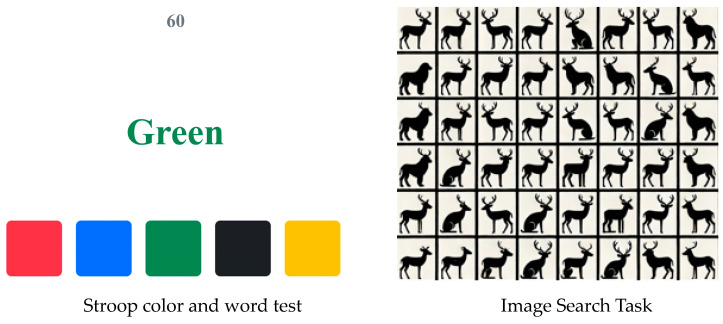
Examples of cognitive tests.

Research has shown that high temperatures can affect human cognitive abilities. When participants answer cognitive questions, their cognitive abilities can be reflected in terms of the time taken and accuracy. Different cognitive abilities can be assessed using various cognitive tests [32,33]. To analyze the effects of temperature and humidity on the cognitive abilities of live-line workers, two cognitive tests were selected: the first test assessed attention and flexibility, while the second tested response and visual perception in terms of executive functions and working memory, measuring reaction speed and accuracy with regard to visual signals. An example of the test is shown in Figure 4. In the Stroop color and word test, participants were required to identify the color described by the word on each page within 60 s, rather than the word itself. In the image search test, participants were asked to locate the specified pattern within a given table. Before the experiment, the participants were instructed on the requirements of the cognitive tests and were asked to practice the tests before the actual experiment.

### 2.6. Statistical Analysis

Statistical analyses were performed using SPSS 27.0 software. The data collected from all participants were analyzed using mean values. The normality of all data was assessed using the Shapiro–Wilk test, and paired t-tests were conducted to analyze the differences in physiological characteristics under different working conditions. One-way analysis of variance (ANOVA) was used to examine the differences in physiological characteristics over time. For all analyses, results with *p* < 0.01 were marked with ‘**’, and results with *p* < 0.05 were marked with ‘*’.

## 3. Results

### 3.1. Core Temperature

**Figure 5:**
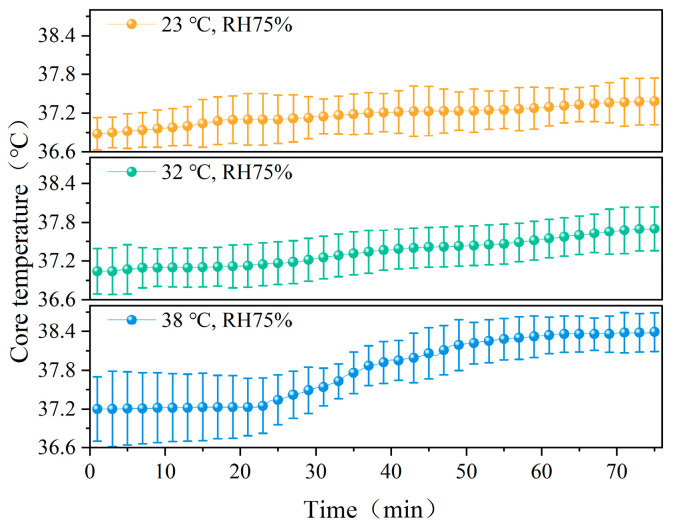
Variation in core temperature with time at different temperatures.

**Figure 6:**
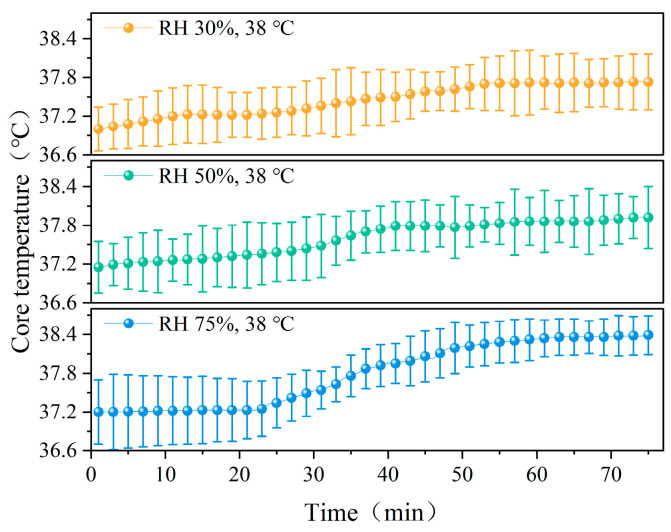
Variation in core temperature with time at different humidity levels.

Figure 5 shows the changes in core temperature over time in participants at different temperatures (23 °C, 32 °C, and 38 °C). As shown in the figure, the core temperature of the participants increased slowly during the first 20 min, then rapidly increased until it stabilized. This may be because participants rested for the first 10 min, during which their metabolic rate was low, and the core temperature began to rise gradually after they started running. A comparison reveals that significant differences in core temperature changes exist among the three temperature conditions (*p* < 0.05). Figure 6 shows the changes in core temperature over time in participants under different humidity conditions (RH 30%, 50%, and 75%). While under RH 75%, the core temperature exhibited the most dramatic changes, with a maximum core temperature of 38.39 °C. Significant differences in core temperature were observed between the three humidity conditions (*p* < 0.05).

### 3.2. Skin Temperature

**Figure 7:**
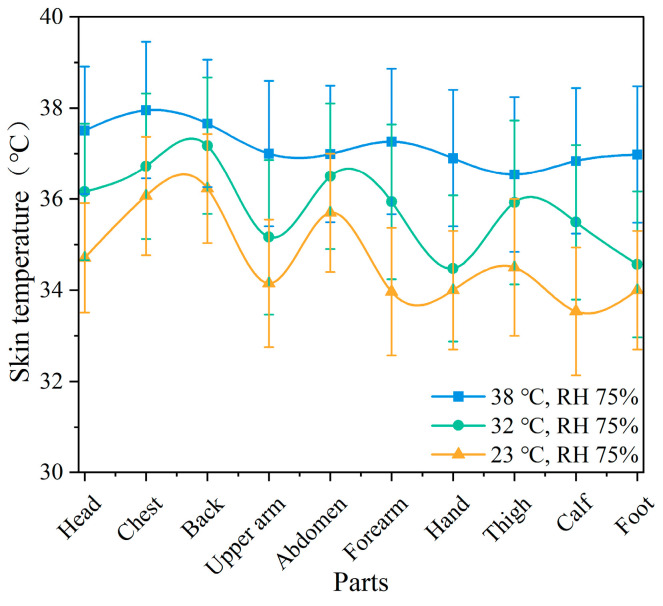
Skin temperature of different parts at different temperatures.

**Figure 8:**
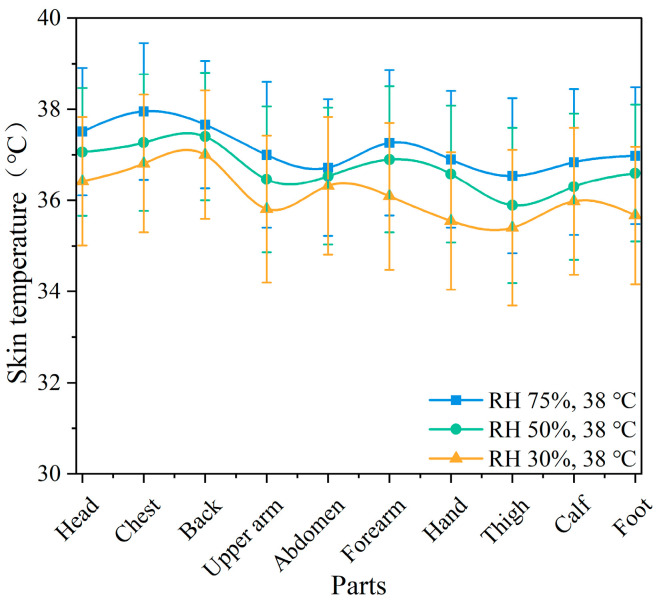
Skin temperature of various parts of the body at different humidity levels.

Figure 7 illustrates the skin temperature of various body parts under different temperature conditions. It can be observed that the skin temperature changes in the back and abdomen were minimal under the three temperature conditions, with no significant differences (*p* > 0.05). The skin temperature of the hands showed the greatest variation (*p* < 0.01). Based on the changes in skin temperature at different body parts, the chest, abdomen, and back showed minimal temperature variation, while the hands, feet, and forearms exhibited greater temperature changes; other body parts experienced moderate variations. As the temperature increased, the differences in skin temperature changes across the body parts decreased, with all body parts reaching higher temperature values. The chest temperature reached a maximum of 37.9 °C; this far exceeds the comfortable skin temperature range of 33–35 °C for the human body. Figure 8 presents the changes in skin temperature at various body parts under different humidity conditions. The temperature changes in the back and abdomen were minimal, with no significant differences (*p* > 0.05). The temperature changes in the hands and feet were more significant as humidity increased, indicating a stronger effect of humidity on distal body parts. The temperature changes in the other body parts were more pronounced compared to the back and abdomen.

The mean skin temperature and standard deviation of the participants at different temperatures are presented in Table 1. As the ambient temperature increased, the mean skin temperature gradually increased. Significant differences were found between the three temperatures (*p* < 0.05). Furthermore, the increase in humidity also led to an elevation in mean skin temperature, though significant differences were observed only between the RH 30% and RH 75% conditions. At the same time, the variation in standard deviation reflects the characteristics of mean skin temperature fluctuations under different conditions. In the low-temperature environment (23 °C), the standard deviation was large (±1.91 °C), indicating significant differences in skin temperature across body parts, which usually occurs when there is a large temperature difference between distal and central body areas. As the temperature increased, the standard deviation gradually decreased, indicating smaller differences in skin temperature across body parts.

### 3.3. Thermal Sensation

**Figure 9:**
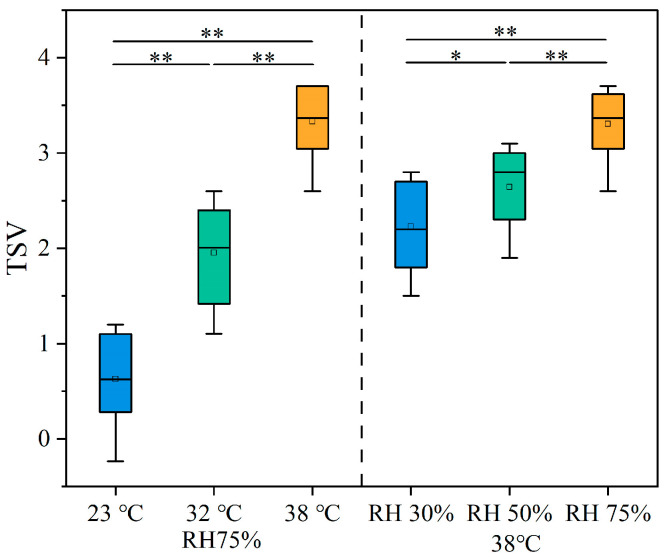
TSV under different working conditions.

The thermal sensation ratings of the participants under different working conditions are shown in Figure 9. As shown in the figure, with the increase in ambient temperature, the participants’ thermal sensation ratings gradually increased. Significant differences in thermal sensation were found among the three temperature conditions (*p* < 0.05). Under the 38 °C condition, the thermal sensation ratings increased to between 2.5 and 4, indicating significant discomfort, and the participants were unable to continue working in this environment. Moreover, the participants’ thermal sensation ratings also showed significant differences with changes in humidity (*p* < 0.05).

### 3.4. Sweat, Heart Rate and Blood Oxygen

**Figure 10:**
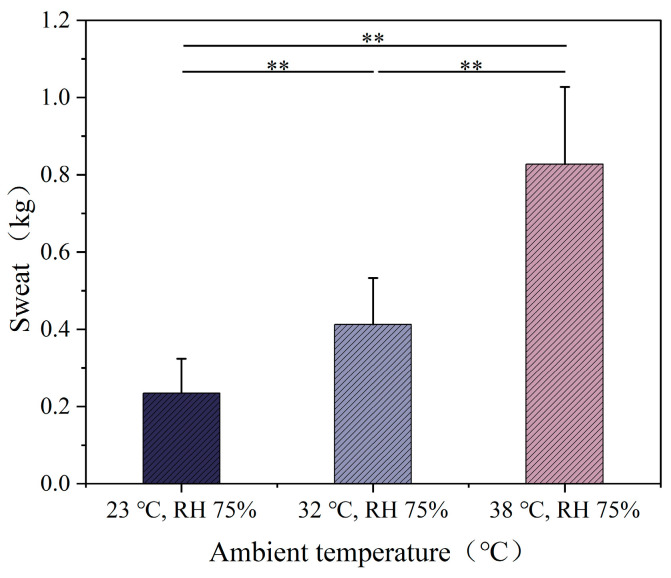
Human sweat volume at different temperatures.

**Figure 11:**
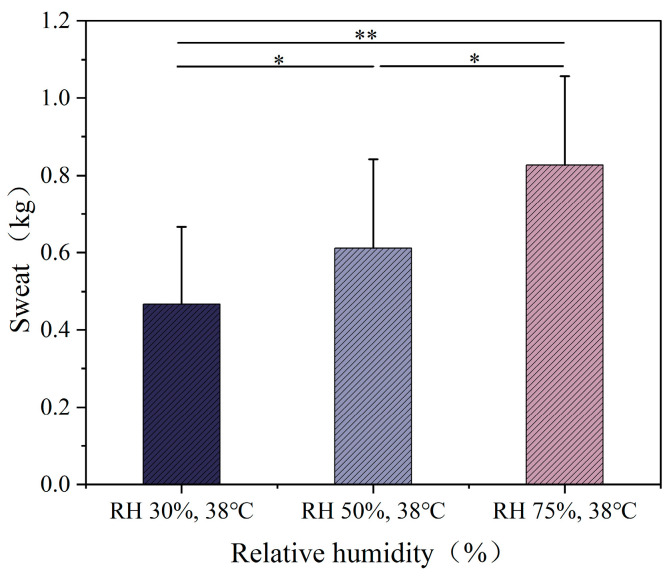
Human sweat volume at different humidity levels.

The sweat rate under different temperature conditions is shown in Figure 10. As the ambient temperature increased, significant differences in sweat rate were observed among the three temperature conditions (*p* < 0.01). Under the 23 °C condition, the sweat rate was relatively low, at 0.234 kg. Under the 32 °C and 38 °C conditions, the sweat rate increased significantly to 0.412 kg and 0.827 kg, respectively, which are 1.8 and 3.5 times the sweat rate at 23 °C. The sweat rate under different humidity conditions is shown in Figure 11. The sweat rate of the participants increased significantly with the increase in humidity. Under the RH 30% condition, the sweat rate was 0.467 kg. Under the RH 50% condition, the sweat rate increased to 0.612 kg. Under the RH 75% condition, the sweat rate increased to 0.827 kg, which is 1.77 times and 1.35 times the sweat rate at the previous two humidity levels, respectively.

**Figure 12:**
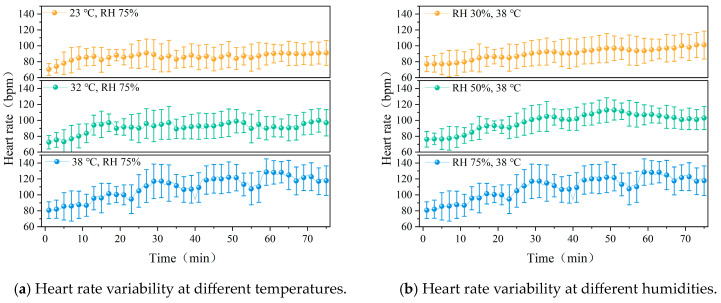
Heart rate over time, under different working conditions.

The trend of heart rate variation in participants under different temperature conditions is shown in Figure 12. As illustrated in Figure 12a, with the increase in environmental temperature, the heart rate of participants exhibited an upward trend, with significant differences in heart rate among the three temperature conditions (*p* < 0.01). As shown in Figure 12b, there were significant differences in heart rate changes under different humidity conditions. As the humidity increased, the amplitude of heart rate fluctuations notably increased. As the environmental temperature and humidity increase, the fluctuation in heart rate gradually intensifies.

**Figure 13:**
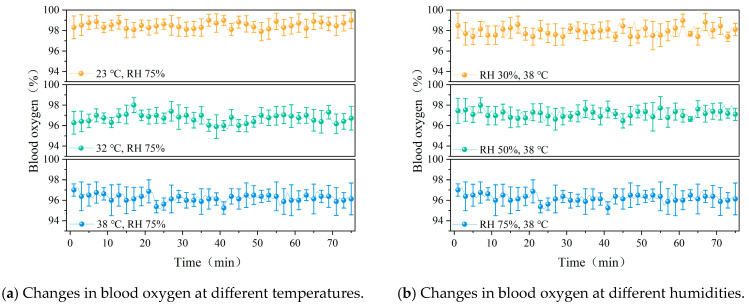
Changes in blood oxygen with time under different working conditions.

The variation in blood oxygen levels under different temperature conditions is shown in Figure 13a. It can be observed that there is no significant difference in blood oxygen saturation between 32 °C and 38 °C (*p* > 0.05). However, blood oxygen levels at 23 °C differ significantly from those at the other two conditions (*p* < 0.05). As shown in the figure, with increasing temperature, blood oxygen levels generally decreased. The variation in blood oxygen under different humidity conditions is presented in Figure 13b. As humidity increased, blood oxygen exhibited a slight downward trend.

### 3.5. Error Rate and Response Time of Cognitive Tests

**Figure 14:**
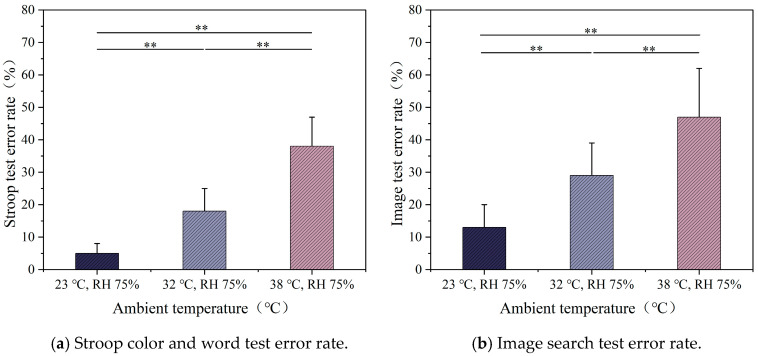
Cognitive test error rates at different temperatures.

Figure 14a illustrates the impact of temperature on the error rate of the Stroop cognitive test. The results indicate that ambient temperature significantly affects the participants’ cognitive test performance. The Stroop test error rate increases with the increases in temperature. Significant differences were found between the three temperatures (*p* < 0.01). Figure 14b shows the impact of temperature on the error rate of the image search test. Similarly to the Stroop test, the error rate in the image search test increased significantly with rising temperature. It is noteworthy that under the same conditions, the error rate of the image search test was higher than that of the Stroop test, and the increase in error rate was more pronounced with rising ambient temperature.

**Figure 15:**
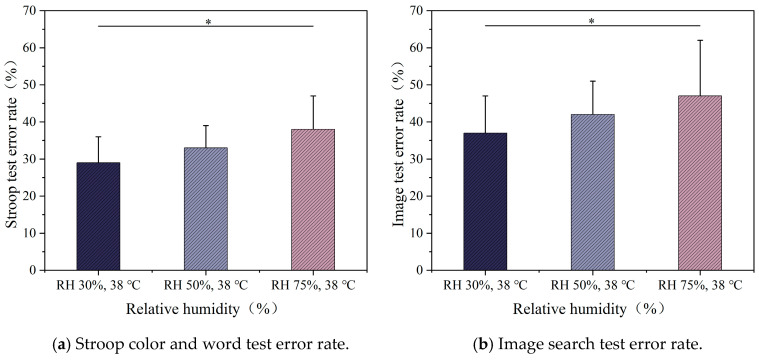
Cognitive test error rates at different humidity levels.

The impacts of different humidity levels on the Stroop cognitive test error rate are shown in Figure 15a. As shown in the figure, significant differences in error rate changes were observed only under RH 75% and RH 30% (*p* < 0.05). Under RH 30%, participants exhibited better cognitive performance, with a lower error rate, whereas under RH 75%, the error rate reached its highest value at 38%. The error rate of the image search test also showed a similar trend (Figure 15b). As humidity increased, the error rate of participants slightly increased, and the rise in humidity had a noticeable negative effect on the performance of the image search task.

**Figure 16:**
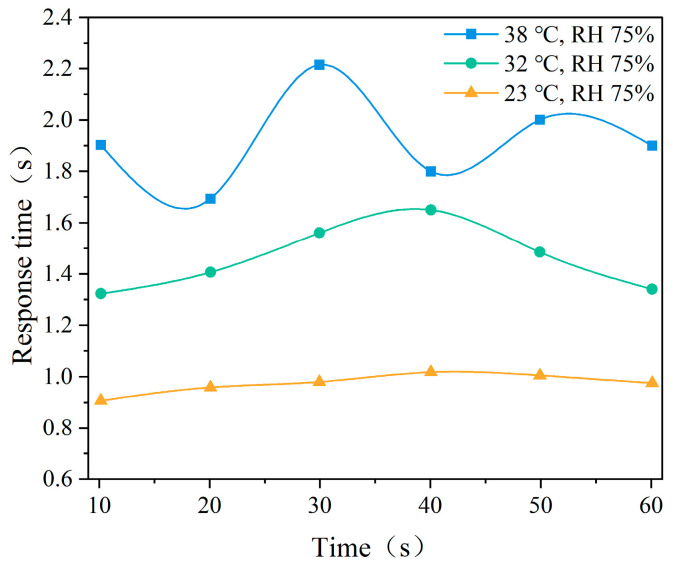
Mean response times at different temperatures.

**Figure 17:**
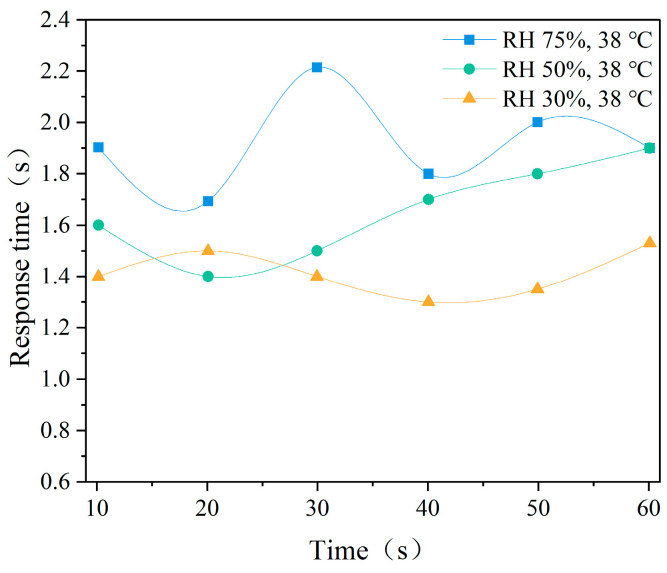
Mean response times at different humidity levels.

Figure 16 presents the average response time of the cognitive test under different temperatures. The results indicate that with an increase in temperature, the average response time significantly increased, with greater variability under high temperature (38 °C), reaching a maximum value close to 2.4 s, reflecting a significant delay in response. The trend of average response time under different humidity conditions is shown in Figure 17. It can be observed that response time increases with increasing humidity.

### 3.6. Variation in Physiological Parameters of the Human Body Under Different Clothing Conditions

**Figure 18:**
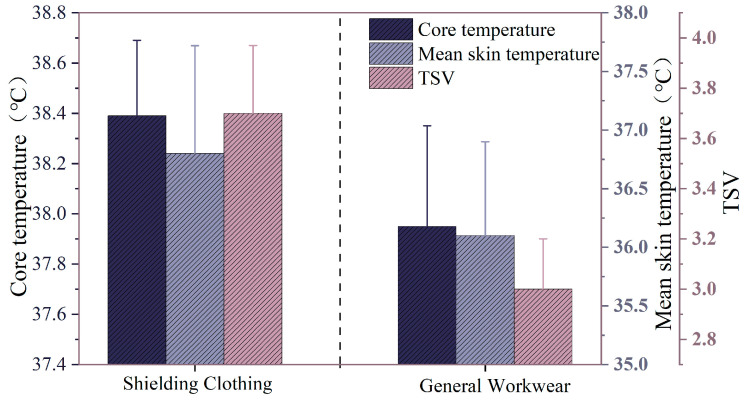
Variation in physiological parameters of the human body with different types of clothing.

Figure 18 presents a comparison of physiological parameters between individuals wearing shielding clothes and ordinary workwear at 38 °C, RH75% environment. As shown in the figure, the core temperature when wearing shielding clothes reached a maximum of 38.39 °C, which is higher than the 37.98 °C recorded for ordinary workwear. The average skin temperature was 36.80 °C for shielding clothes, compared to 36.10 °C for ordinary workwear. Additionally, the thermal sensation score was 3.7 when wearing shielding clothes, as opposed to 3.0 for ordinary workwear. The CHSI values were 5.1 ± 0.2 when wearing shielding clothes, as opposed to 4.2 ± 0.1 for ordinary workwear. This suggests that shielding clothes may inhibit the body’s heat dissipation, leading to increased thermal accumulation, which in turn results in higher core and skin temperatures compared to ordinary workwear. The increased thermal load may be related to the moisture permeability and breathability of the shielding clothes material. Furthermore, the higher thermal sensation score under the shielding clothes condition reflects an enhanced participative feeling of heat discomfort, consistent with the trend observed in temperature data. Therefore, shielding clothes in high temperature and humidity environments may increase the thermal physiological burden of individuals performing live-line work. Effective cooling measures are urgently needed to ensure their occupational safety and work efficiency.

## 4. Discussion

### 4.1. Effect of Different Temperatures and Humidity on Body Temperature

**Figure 19:**
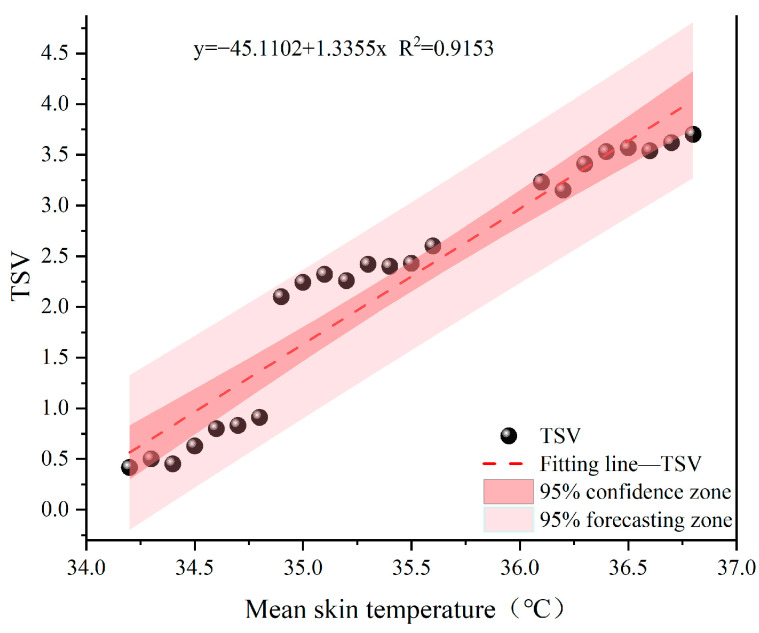
Relationship between thermal sensation and mean skin temperature.

Previous studies have shown that in high-temperature and high-humidity environments, the core body temperature significantly increases, leading to increased heat stress load [34]. In our study, the maximum core temperatures in the 32 °C and 38 °C environments were 37.8 °C and 38.4 °C, respectively. Under similar temperature and humidity conditions, studies involving lower thermal resistance clothing have found core temperatures lower than those observed in our study [26,30,31,35]. Furthermore, our study compared the thermal physiological responses of participants wearing shielding clothing versus ordinary clothing, The CHSI values were 5.1 ± 0.2 when wearing shielding clothes, as opposed to 4.2 ± 0.1 for ordinary workwear, confirming that electrical grid workers in shielding clothing are more prone to heat stress reactions than those wearing regular work attire. Shi et al. [36] pointed out that the combined effects of temperature and humidity are more significant than the individual effects of either factor, especially when humidity is high. Under these conditions, the body’s thermoregulatory function is limited, and sweat evaporation is reduced, leading to a significant increase in core temperature. In this study, changes in skin temperature increased with rising temperature and humidity. Notably, at the extremities, such as the hands and feet, the temperature change was more pronounced, possibly due to reduced blood supply and heightened sensitivity to heat stress at these sites. This trend aligns with previous research [37,38]. It has been reported that skin temperature can represent thermal sensation in a uniform thermal environment [39,40]. Thus, this study also analyzed the relationship between average skin temperature and thermal sensation in high humidity environments. As shown in Figure 19, in real-world scenarios, obtaining average skin temperature can be complex and difficult due to various limitations. Therefore, thermal sensation can be used as a physiological warning indicator [41]. A fitting formula for thermal sensation and average skin temperature was established to predict the average skin temperature in high humidity environments. When the thermal sensation is −0.5, 0, and +0.5, the corresponding average skin temperatures for neutral thermal comfort were found to be 33.4 °C, 33.8 °C, and 34.1 °C, respectively. When thermal sensation falls within the −0.5 to +0.5 range, the comfortable range for average skin temperature is approximately 33.4–34.1 °C. Compared with the studies of Liu et al. [37] and Zhou et al. [40], this study found a broader comfortable skin temperature range, as they reported that the comfortable range for average skin temperature was 32.6–33.7 °C and 32.3–33.9 °C, respectively. This discrepancy may be due to the increased heat tolerance of the body in high-temperature, high-humidity environments, enabling the body to better cope with the heat discomfort caused by such environments.

### 4.2. Effect of Different Temperature and Humidity on Physiological Responses

**Figure 20:**
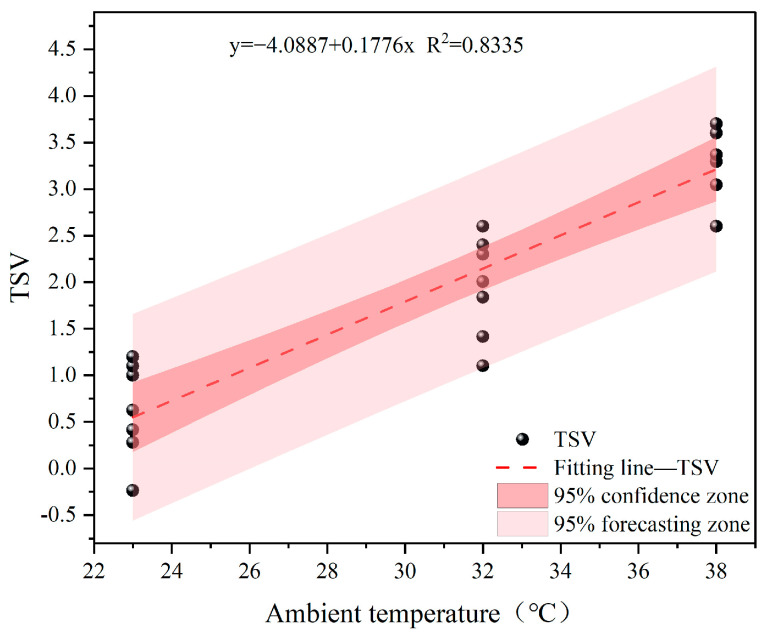
Relationship between ambient temperature and thermal sensation.

Jin [42] demonstrated that when the ambient temperature (Ta) was 32 °C, significant changes in the human thermal response occurred as the humidity increased from 70% to 90%. However, when the humidity increased from 50% to 70%, only the sensation of humidity and skin wetness showed significant changes. Earlier, Ichi et al. [43] and Leow et al. [44] reported that when the humidity was below 70%, its effect on thermal sensation was not significant. However, this study found that there were also significant differences in the effect on thermal sensation when humidity was below 70%. As temperature and humidity increased, the thermal sensation scores of the participants significantly increased. This indicates that under high-temperature and high-humidity conditions, the thermal discomfort of participants wearing shielding clothing was more intense. Moreover, this study analyzed the variation in thermal sensation voting (TSV) at different temperatures and proposed a linear regression equation (Figure 20). This equation can predict the optimal thermal comfort temperature for humans and provide a reference for indoor environmental temperature control. When TSV is equal to −0.5, 0, and +0.5, the corresponding environmental temperatures for neutral thermal sensation were calculated to be 20.77 °C, 23.02 °C, and 25.84 °C, respectively. Thus, when the environmental temperature falls within the range of 20.8–25.8 °C, the TSV will be between −0.5 and +0.5. According to Fang et al.’s research, the neutral temperature at normal pressure is approximately 24 °C, and when the TSV range is between −0.5 and +0.5, the comfort range is between 21.56 °C and 26.75 °C [45]. Additionally, the ASHRAE comfort range is 23.0–27.0 °C [46]. Compared to previous studies, our study found that the comfort range for the participants was narrower, suggesting that the comfort temperature for human participants may decrease in high-humidity environments.

Under 32 °C and 38 °C conditions, volume increased to 0.412 kg and 0.827 kg, respectively, which were 1.8 and 3.5 times higher than in the 23 °C environment. This indicates that high temperatures, combined with the wearing of shielding clothing, significantly elevate volume. In high-humidity environments, volume further increased, suggesting that humidity limits sweat evaporation, prompting the body to intensify sweating. As temperature and humidity rise, heart rate also increases, likely due to the limited evaporation of sweat and the increased difficulty for heat dissipation, which places greater strain on the cardiovascular system. Fitzgerald et al. [47] and Nag et al. [48] observed similar trends in heart rate under high temperatures, and our study found that shielding clothing exacerbates this response, increasing thermoregulatory and cardiovascular function loads.

While previous research explored the effects of temperature and humidity on thermal responses, our study’s focus on live-line workers wearing shielding clothing under high-temperature and high-humidity conditions provides new insights into how environmental factors interact with protective gear to influence worker comfort and safety. Our findings highlight the need for tailored thermal management strategies in the design of protective clothing for high-risk occupations, and suggest that improving comfort in such extreme environments may require innovations in both clothing materials and environmental control systems.

### 4.3. Effects of Different Temperatures and Humidity on Cognitive Performance

Our study found that as temperature and humidity increased, participants exhibited significantly higher error rates and longer reaction times, indicating that high temperature and humidity not only increase physiological discomfort but also impair cognitive performance. Specifically, in high-humidity conditions (75%), cognitive response speed and accuracy were reduced, with error rates increasing more noticeably in tasks that required higher cognitive control, such as the image search task. This suggests that the complexity of cognitive tasks plays a significant role in how environmental stressors, such as heat and humidity, affect performance. The Stroop test also showed that higher temperatures caused greater cognitive interference, but the image search task demonstrated a more pronounced increase in error rates, reflecting the higher cognitive demands of the task.

This study’s findings provide new insights into the effects of high temperature and humidity on cognitive performance in live-line workers wearing shielding clothing. Unlike previous studies that focused on general work environments, our research specifically addressed the thermal and cognitive responses of individuals in high-temperature, high-humidity conditions while wearing shielding clothing. This setting is more representative of the conditions experienced by live-line workers and highlights the compounded effects of shielding clothing, which may further exacerbate the impact of environmental stressors on cognitive performance and decision-making. The results underscore the importance of considering both environmental factors and personal protective equipment in designing safe and effective work conditions for workers exposed to extreme temperatures and humidity.

### 4.4. Limitations and Future Study

This study reveals human responses to temperature and physiological reactions in high-temperature and high-humidity environments. Nevertheless, there are some limitations to this study. Specifically, the participants were recruited as healthy young male individuals according to the standards for live-line workers. Furthermore, considering the level of adaptation to previous living environments, studying human physiological responses in high-temperature and high-humidity settings would be more beneficial. Additionally, incorporating a wider range of temperature and humidity conditions, as well as different age groups, would help to draw more valuable conclusions. Our laboratory is continuing further research. More importantly, future studies should focus on the development of personal thermal protective equipment, such as the design of new personal cooling garments, to ensure the safety and work efficiency of live-line workers in high temperature and humidity environments.

## 5. Conclusions

This study reveals the significant impact of various temperature and humidity conditions on the physiological and cognitive abilities of live-line workers, and analyzes the severity of heat stress issues faced in high-temperature and high-humidity environments from multiple perspectives. The main conclusions are as follows:

(1) With the increase in temperature and humidity, the core temperature, heart rate, and thermal sensation scores of participants wearing high-voltage-shielding clothing significantly increased, and were higher compared to participants wearing standard workwear. In cognitive testing, high temperature and humidity conditions significantly reduce task accuracy and response speed. Moreover, more complex tasks exhibit even more significant negative effects.
(2) A heat sensation prediction model for live-line workers based on environmental temperature under high humidity conditions was established to predict human heat sensation through environmental temperature. In the neutral heat sensation range (−0.5~+0.5), the environmental temperature should be within the range of 20.8–25.8 °C.
(3) Another prediction model for heat sensation is based on the average skin temperature under high humidity, aimed at predicting the average skin temperature through human heat sensation. In the neutral heat sensation range (−0.5~+0.5), the average skin temperature should fall within the range of 33.4–34.1 °C.
(4) The shielding clothing significantly inhibits the heat dissipation of the participants, leading to increased heat accumulation within the body compared to normal work clothing. Consequently, live-line workers face higher physiological and cognitive burdens. Therefore, effective cooling measures are urgently needed to ensure their occupational safety and work efficiency. The results of this study provide a theoretical basis for the development of personal protective equipment for live-line workers.

## Figures and Tables

**Table 1:** Average skin temperature under different operating conditions.

	Ambient Temperature			Relative Humidity		
	RH 75%				38 °C	
Experimental conditions	23 °C	32 °C	38 °C	RH 30%	RH 50%	RH 75%
Average skin temperature (°C)	34.8	35.6	36.8	36.1	36.4	36.8
Standard deviation (°C)	±1.91	±1.23	±0.92	±1.12	±0.99	±0.92

## Data Availability

Data will be made available on request.
